# Examining Relationships between Cognitive Flexibility, Exercise Perceptions, and Cardiovascular Disease Risk Factors

**DOI:** 10.3390/ejihpe13100161

**Published:** 2023-10-17

**Authors:** Bryan M. Buechner, Miranda K. Traylor, Rachel I. Feldman, Kaitlyn F. Overstreet, Benjamin D. Hill, Joshua L. Keller

**Affiliations:** 1 Williams College of Business, Xavier University, 3718 Francis Xavier Way, Cincinnati, OH 45207, USA; 2 Integrative Laboratory of Exercise and Applied Physiology (iLEAP), Department of Health, Kinesiology, and Sport, College of Education and Professional Studies, University of South Alabama, 171 Student Services Dr, Mobile, AL 36688, USA; mkt1621@jagmail.southalabama.edu (M.K.T.);; 3 Department of Psychology, College of Arts and Sciences, University of South Alabama, Humanities Room 118, Mobile, AL 36688, USA; bdhill@southalabama.edu

**Keywords:** exercise adherence, cognitive flexibility, exercise, cardiovascular disease, modifiable risk factors, management of health risks

## Abstract

Adults do not engage in enough physical activity. Investigating cognitive and physiological factors related to improving this behavior—and reducing health risks—remains a public health priority. Our objective was to assess whether cognitive flexibility influenced perceptions and choice of exercise programs and whether flexibility was associated with cardiovascular disease (CVD) risk factors. Independent sample groups of college-aged adults (18–24 yrs) participated in two studies. Data were collected on individuals’ degree of cognitive flexibility (both self-reported and objectively measured), perceptions and choice of exercise programs, and health status markers known to be associated with CVD (vascular function, muscular strength, and body composition). Vascular function was assessed with a near-infrared spectroscopy device, strength was defined as handgrip, and body composition was estimated via digital circumferences. Self-reported flexibility reliably predicted individuals’ choice of exercise program and perceptions of effort required for success on an exercise program. The relationships among CVD risk factors and objectively measured cognitive flexibility were not significant, demonstrating that identifying a healthy individual’s degree of performance-based cognitive flexibility does not predict health status. Furthermore, although greater self-reported trait flexibility (rigidity) is known to predict higher (lower) likelihood of physical activity, this finding should not be extrapolated to also assume that flexibility (rigidity), as measured by objective cognitive tests, is associated with reduced CVD risk in healthy adults. We posit a rationale for how understanding cognitive flexibility and rigidity can play an impactful role in improving adherence to exercise prescriptions targeted to reducing risks.

## 1. Introduction

There is a well-accepted bidirectional relationship between behavior and physiology, which is generally included within the field of psychobiology. This is demonstrated by reports that sedentary individuals have a higher risk for developing cardiovascular disease (CVD), as well as that various diseases and conditions may cause individuals to avoid healthy behaviors such as participating in regular exercise [1,2,3,4,5,6,7,8,9,10,11,12]. Adhering to exercise is a well-established strategy to promote physical and cognitive health [2,3,4,6,11,12,13]. Yet, most adults in the United States (US) do not engage in adequate levels of physical activity and exercise [14] or successfully adhere to programs designed to improve their physical health [15,16,17]. For example, only about one half of US adults meet physical activity guidelines, but less than 25% actually complete aerobic and muscle-strengthening activities [14]. As well as reports of lack of time, a top determinant of exercise intervention dropout includes a lack of motivation. Unfortunately, to date and almost exclusively, the previous research studies that aimed at addressing the widespread underachievement in physical activity levels while considering psychological aspects like cognition have done so by examining the relationships between overall cognitive and health status [9,18,19]. For instance, Brush et al. [19] reported that cardiorespiratory fitness positively correlated with cognitive ability, and further, it was shown that individuals presenting with the lowest baseline cardiorespiratory fitness experienced the greatest decline in the Mini-Mental State Examination (MMSE; an assessment of global cognitive function) in response to aging [18]. To be clear, much like MMSE scores, cardiorespiratory fitness (e.g., VO_2max_) is a comprehensive measurement that encompasses many organ systems and physiological processes, and thus this measure has limited precision. Therefore, there is a critical need to evaluate specific domains of cognition and measures of health, so that future clinical trials and interventions may better directed and more targeted. 

A meaningful cognitive domain to examine is cognitive flexibility, due to the emerging evidence that has focused on the influence of cognitive flexibility on healthy behaviors, especially relating to physical activity [20,21,22,23]. Cognitive flexibility facilitates important human functioning, such as modifying one’s behavior to changing or complex circumstances or task demands [24]. Individuals vary in their degree of flexibility; cognitive rigidity (e.g., the tendency to be inflexible in thought) may be considered the opposite [25,26], and various definitions have been proposed to capture this trait of cognition and behavior [25]. Practical applications include reports that cognitively flexible individuals test new rules when learning and explore multiple strategies when problem solving, whereas cognitively rigid individuals learn through perseverance, and exploit active strategies at the expense of alternatives when problem solving [27]. Further, flexible (rigid) individuals tend to seek out learning experiences that deepen (broaden) their knowledge of a topic [28,29]. To summarize, individuals seem to intuitively choose experiences that align with their level of cognitive flexibility. 

However, we are currently unaware of whether this notion has been applied to exercise programming and/or the resultant health outcomes. This is surprising, given that cognitive flexibility influences self-control-related behaviors like planning for the future and achieving defined goals [30,31]. Cognitive flexibility likely plays a critical role in influencing healthy behavior, which is well known to require self-control in goal pursuit, especially when the goal has less constraints and a less defined path forward [5,7,27,32,33]. However, Buechner et al. [34] previously reported that successful self-control can be contingent upon a match between one’s degree of cognitive flexibility (rigidity) and the demands of a given situation; when there is a mismatch, self-control performance decreases. This conflict may induce a type of proactive interference, which can increase the (perceived) effort needed to complete a task and simultaneously cause a decrease in performance [35,36]. Although flexibility may be beneficial in contexts where task demands change (e.g., a “match” [5,7]), research is needed to identify how individuals perceive certain exercise programs when there is a mismatch. In theory, if program demands do not match an individual’s flexibility, then the program may appear to require more effort to be effective, which could undermine performance, ultimately leading to unchanged, or even worse, health outcomes. This mismatch may also lead to further declines in motivation and overall enjoyment in exercise.

Cognitive flexibility may serve as a predictor of willingness to engage in different types of physical activity and exercise [5,33], and thus it is rational to suspect it to be linked to lower CVD risk [23]. However, to date, there are only minimal data describing the relationships between unique markers of cognition (e.g., cognitive flexibility) and precise markers of health. Thus, additional data are needed to identify relationships between levels of cognitive flexibility and practical, novel markers of health status. Fruitful measures of health likely include those that characterize CVD risk, such as measures of peripheral vascular function [37,38], handgrip strength, and body composition [39,40]. Despite the ability of these health markers to predict CVD and the importance of cognitive flexibility in promoting healthy behavior, no previous study has investigated the existence of a direct, causal relationship among them. Furthermore, based on the lack of currently available data, it is worthy of investigation to determine whether—within a healthy population—there is a significant relationship between CVD risk and cognitive flexibility.

Therefore, the purpose of the current investigation was to investigate the influence of cognitive flexibility (using both self-reporting and objective measures of cognitive flexibility) on perceptions and choice of exercise programs and the potential causal relationship between cognitive flexibility and specific markers of health (vascular function, handgrip strength, and body composition). Based on previous findings, it was hypothesized that: (i) when given a choice of health and wellness programs, individuals’ level of flexibility will influence their preference, such that their choice is a match between their flexibility and the structure of the program; (ii) individuals’ cognitive flexibility will influence perceptions of exercise programs, such that when flexibility mismatches the demands of a program, individuals will perceive the program as requiring more effort to be effective; and (iii) cognitive flexibility will be predictive of health markers. 

## 2. Materials and Methods

### 2.1. Ethical Approval

The collection of human data was performed according to the ethics standards established by the Declaration of Helsinki, 2013 (except for preregistering in a publicly accessible database before the recruitment of the first participant) and was approved by the local Institutional Review Board for Human Subjects at the authors’ respective institutions. All participants gave informed consent prior to the completion of any experiment.

### 2.2. Human Participants and Power Analysis

Independent sample groups of college-aged adults participated in two discrete studies. For Study 1, 254 college-aged adults were recruited from a university setting (M_age_ = 20, SD_age_ = 1.9 yrs; 51% male) and volunteered to complete a survey questionnaire of self-reported cognitive flexibility. For Study 2, 30 healthy college-aged adults (M_age_ = 22, SD_age_ = 1 yr; 50% male) were enrolled to complete two in-person, physiological testing visits and an objective performance-based measure of cognitive flexibility. A priori power analyses (correlation effect: size ρ = 0.5, α = 0.05, power = 0.8) were used to determine the sample sizes for each study. For Study 1, a total, minimal sample size of 148 was recommended [41]. For Study 2, based on previous findings [42,43], a total, minimal sample size of 26–29 was recommended, which was reached with 15 men and 15 women (n = 30). 

### 2.3. Procedure

In Study 1, participants made a choice between virtual fitness programs and healthy cooking classes. Based on previous research, individuals have several options when choosing health improvement programs, and many include emphasis on both diet and exercise [16]. Specifically, participants were presented with (in a random order) a hypothetical choice between two virtual fitness classes; one class was framed to be rigid (e.g., “this class requires a strict routine”) while the other was framed to be flexible (e.g., “this class requires variation”). Participants made a similar choice between two healthy cooking plans; one plan was framed to be rigid (e.g., “program utilizes structured cooking methods and exploits one perspective for healthy eating”), while the other was framed to be flexible (e.g., “program utilizes multiple cooking methods and integrates different perspectives on healthy eating”). Critically, each choice set contained a no-choice option [44]. Participants who chose “neither” were excluded from analysis (N = 18 for fitness choice; N = 12 for cooking choice). Next, participants evaluated a commercial exercise program (BeachBody On Demand). Participants read a description of the program, a fictitious print advertisement for the program, and customer reviews of the program. Critically, the program was manipulated to be rigid (e.g., “this program has a clear regimen”, “we tell you exactly what to eat to lose weight”); thus, participants assessed the program’s flexibility on two 9 pt scales anchored from structured/rigid to unstructured/flexible (*r* = 0.581, *p* < 0.001). This served as our manipulation check. Then, they indicated how much effort they would need to exert to be successful with the program and the overall perceived effectiveness of the program on 9 pt scales anchored from No effort at all/Not at all effective to A great deal of effort/Very effective. Next, participants completed a self-report measure of cognitive flexibility, which likely captured the personality trait aspect of this specific cognitive domain. Lastly, participants reported their demographic information (e.g., age, sex) and were thanked for their time and participation.

In Study 2, participants visited the Integrative Laboratory of Exercise and Applied Physiology (iLEAP) at the University of South Alabama on two occasions to complete assessments of peripheral vascular function, handgrip strength, and body composition. Participants also completed a computer-based measurement of cognitive flexibility performance in a quiet, empty room in the laboratory on each visit. Of note, unlike Study 1, this was an actual objective of cognitive flexibility. Prior to completing their physiological assessment, the participants completed a self-reported health history questionnaire to verify that they had no known cardiovascular, metabolic, musculoskeletal, or renal diseases.

### 2.4. Measures and Analyses

#### 2.4.1. Cognitive Flexibility

Cognitive flexibility is a complex property of cognition, comprised of several executive functions that are typically assessed using multiple methodologies (e.g., self-report, objective, measures, etc.), and the extant research recommends using multiple methods when assessing flexibility to provide a comprehensive view of the construct [25]. To achieve this aim, in Study 1, we utilize the Actively Open-Minded Thinking Scale (AOT) due to its documented usage as a self-report measure for cognitive flexibility [45,46]. The AOT includes items such as “People should revise their beliefs in response to new information or evidence”, and responses are scored on 7 pt scales anchored from Completely disagree to Completely agree. Higher (lower) scores on this scale suggest greater flexibility (rigidity). 

In Study 2, participants completed the CNS Vital Signs assessment (FDA Medical Device Registration Number: 3006559064), which is a well-accepted neurocognitive battery that encompasses numerous different tests. The tests available in the CNS Vital Signs assessment have been reported to be valid and reliable, as well as sensitive to small changes in performance [7,47,48]. Specifically, from the available tests, the current participants completed the Shifting Attention Test (SAT) and the Stroop Test. The SAT assessed the ability of an individual to shift from one instruction set to another, such as under the current conditions, which included asking participants to randomly match geometric objects by color or shape [48]. This test may be viewed as similar to the shifting attention requirements of more traditional tests such as Trail B and the Wisconsin Cart Sort. Here, however, the cognitive flexibility score was generated from the results of the currently used tests by subtracting the number of errors found on the SAT and Stroop Test from the total number of correct responses during the SAT [7,25,48]. This method of calculating cognitive flexibility has previously been highlighted by Ionescu [25], and includes conceptualizing cognitive flexibility as a high-order ability including cognitive control. 

#### 2.4.2. Peripheral Vascular Function

**Figure 1:**
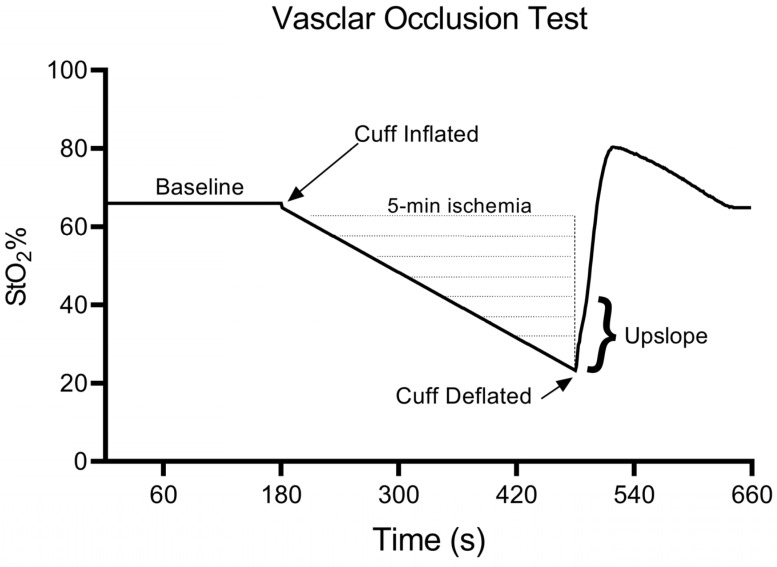
This schematic depicts the timeline of events during the vascular occlusion test (VOT), such that baseline tissue oxygenation (StO2) is quantified for 3 min before blood flow to the forearm muscular is completely occluded for a duration of 5 min. At the 5 min timepoint, the blood flow is rapidly restored, and the rate of restoration is quantified as the linear slope coefficient (b) across the initial 10 s of reperfusion. Recovery is assessed for a duration of 3 min following the deflation of the cuff, which results in a total VOT time of 11 min.

Reactive hyperemia is a common physiological response used to characterize microvascular function within the peripheral vascular system and to quantify CVD risk [37,38]. To assess reactive hyperemia, a near-infrared spectroscopy device was used during a vascular occlusion test (VOT) [49]. Our protocol consisted of each participant resting for 5 min on a standard treatment table. During the rest, two blood pressure (BP) cuffs were attached around the right arm. Subsequently, ultrasound images of the right forearm were collected to ensure that less than 2 cm of adipose tissue was beneath the device (ultrasound-derived adipose tissue thickness: M_males_ = 0.44, SD = 0.2 cm; M_females_ = 0.60, SD = 0.1 cm). The device provided relative changes with respect to the initial baseline value in skeletal muscle tissue oxygenation (StO_2_, %). Once the ultrasound images had been taken, the NIRS device was attached. Specifically, our VOT matched the methodology in the relevant literature [38,49], as depicted in Figure 1.

Once the rest period was over, the upper and lower BP cuffs were simultaneously and rapidly (<0.3 s) inflated to a suprasystolic value (~200 mmHg) and these cuffs were automated by two identical devices (E20 Rapid Cuff Inflator, D. E. Hokanson, Inc., 12840 NE 21st Place, Bellevue, WA 98005, USA). At the level of the upper arm and wrist, the total blood flow occlusion (i.e., transient ischemia) was maintained for 5 min while the NIRS device continuously recorded StO_2_. After 5 min of transient ischemia, only the upper cuff was deflated, which induced reactive hyperemia while preventing blood from pooling in the hand vasculature. During the VOTs, the continuous assessment of StO_2_ allowed for the determination of the upslope (i.e., rate of reperfusion). This upslope value was defined as the linear change in the StO_2_ value following the 5 min of ischemia and used as a marker of reactive hyperemia [38,49], and thus, peripheral vascular function (Figure 1).

#### 2.4.3. Handgrip Strength 

The participants were guided through a standardized warm-up consisting of separate handgrip muscle actions at estimated intensities of 25%, 50%, and 75% of their maximal voluntary isometric contraction (MVIC), that is, a maximal effort squeeze. Once adequately familiarized with the handgrip dynamometer (microFET Handgrip, Hoggan Scientific, LLC, Salt Lake City, UT, USA), the participants completed 2–3 dominant hand attempts of 6 s MVIC trials. A third trial was only used if the force produced during MVIC trials 1 and 2 varied by ≥5%. Strong verbal encouragement was given during each trial, and the participants were able to view the real-time force value.

#### 2.4.4. Body Composition 

The body composition of each participant was measured using a Fit3D ProScanner (Redwood City, CA, USA) in accordance with the manufacturer’s instructions. This included instructing the participants to stand on a turntable and grasp handles at the sides, such that the arms would be fully extended and slightly abducted. Once they were in the correct posture, the turntable slowly rotated, with the scanner moving up and down to rapidly collect images. A 3D digital image was generated from the measured circumferences, heights, lengths, and widths, which allowed for an estimate of the lean and fat tissue quantities (kg). For proper, accurate assessment, each participant wore minimum, form-fitting clothing. 

### 2.5. Data and Statistical Analyses

All analyses were conducted using Statistical Package for the Social Sciences software (version 28.0 Chicago, IL, USA). Tests for normality and outliers were conducted prior to the defined statistical tests below. There were no missing pairs or violations of the assumptions of correlation. Zero-order Pearson’s correlations (*r*), logistic regression, linear regression, and statistical modeling were all used to determine the relationships between cognitive flexibility and perceptions of exercise programs; 95% confidence intervals (CI) were used for correlational analyses and for tests of mean differences, and a *p*-value ≤ 0.05 was considered statistically significant for all results.

CNS Vital Signs normed the scored used for analysis with a mean score of 100 and a standard deviation of 15, with higher scores indicating better performance (i.e., greater flexibility). The cognitive flexibility values used for analyses were the standard scores, which were the normalized raw scores representing an age-matched value relative to other individuals in a normative sample. 

Peripheral vascular function was defined as the rate of reperfusion and determined via simple linear regression to derive the StO_2_ vs. time slope coefficient for each participant. Analysis of handgrip strength was completed using the maximal value observed during 2–3 handgrip MVICs. In addition to absolute body composition values (kg of fat and lean mass), relative body composition was calculated as percent body fat (fat mass ∙ total body mass^−1^ ∙ 100). These values were recorded during both visits associated with Study 2, which allowed for an average of each health parameter to be determined for each participant in attempt to analyze stable characteristics. For all the health status parameters (peripheral vascular function, handgrip strength, and body composition) collected during Study 2, the test–retest reliability was calculated as the 2,1 model intraclass correlation coefficient (ICC_2,1_). To provide additional support for the validity of our physiological measures, mean sex differences among the health status parameters were tested via independent *t*-tests. 

## 3. Results

### 3.1. Study 1

The objective of Study 1 was to assess individuals’ perceptions of different programs aimed at improving one’s health. Correlation analysis revealed that individuals’ cognitive flexibility (i.e., AOT; α = 0.67) significantly correlated with the choice of the flexible fitness class (*r* = 0.132; *p* = 0.04, 95% CI [0.010, 0.252]) and the choice of the flexible cooking plan (*r* = 0.134, *p* = 0.04, 95% CI [0.008, 0.256]). There were no significant correlations between cognitive flexibility and the sex of the participant (Table 1).

The choice data (contrast coded; −1 = rigid choice, 1 = flexible choice) were submitted to a logistic regression, with cognitive flexibility (continuous, mean-centered) as the predictor. The results revealed a main effect of cognitive flexibility for the fitness class (B = 0.42, Wald’s χ^2^ = 4.08, *p* = 0.04, 95% CI [1.012, 2.282]) and cooking program (B = 0.45, Wald’s χ^2^ = 4.17, *p* = 0.04, 95% CI [1.018, 2.403]). Individuals with higher flexibility were more likely to choose the flexible (vs. rigid) option. 

**Figure 2:**
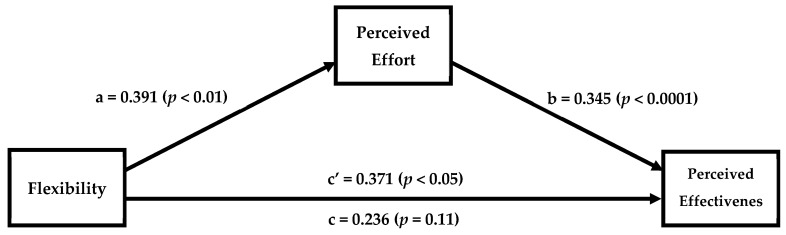
Study 1. Effect of flexibility on perceptions of an exercise program’s effectiveness through perceived effort.

To check our manipulation of the commercial exercise program, a one-sample *t*-test with the scale midpoint (5) as the test value was used to assess whether the program’s structure was best defined as “rigid” or “flexible”. The mean rating (M = 3.65) of the items assessing the program’s flexibility was significantly different to the midpoint (*t* = −10.870; *df* = 253; *p* < 0.001; 95% CI [−1.593, −1.104]), suggesting that all participants perceived Beachbody as being a rigid (vs. flexible) program. Linear regression analyses supported that individuals’ flexibility influenced their perception of the effort needed in the program (B *=* 0.390; SE = 0.141, *p* < 0.01, 95% CI [0.112, 0.669]) and the effectiveness of the program (B *=* 0.371; SE = 0.154, *p* = 0.02, 95% CI [0.068, 0.675]); as flexibility increased, so did the perception of the effort needed and the perceived effectiveness of the program. Using bootstrapping procedures [50], we tested the mediating role of perceived effort by computing a 95% CI around the effect of flexibility on perceived efficacy through the proposed mediator (Model 4). The mediation pathway through perceived effort was significant (indirect effect: 0.0411; 95% CI [0.034, 0.253]; see Figure 2). Individuals with higher degrees of cognitive flexibility perceived the program to require more effort (due to a mismatch), whereas individuals with lower degrees of cognitive flexibility perceived the program to require less effort (due to a match). Participants also reported their sex and age (M/age = 20, SD/age = 1.9 yrs; 51% male). Including these variables as covariates in the mediation model did not significantly alter the results; the mediation pathway through perceived effort was significant (indirect effect: 0.1406; 95% CI: 0.030, 0.269).

### 3.2. Study 2

The objective of Study 2 was to identify any potential relationships between cognitive flexibility and CVD risk factors. There were no observed significant (*p* > 0.05) relationships among the health status parameters and measures of cognitive flexibility derived from the CNS Vital Signs. All means are reported in Table 2 and a full description of correlations for Study 2 measures can be found in Table 3. Additionally, sex did not significantly correlate with our measure of cognitive flexibility (derived from the CNS VS), and these results are reported in Table 1.

Study 2 also provided results to support the interpretations derived from the above results. For example, analysis revealed suitable test–retest reliability (ICC_2,1_ = 0.56–0.98), the repeated measures ANOVA indicated no significant systematic error (*p* = 0.06–0.19) (for exact values, see Table 4), and independent *t*-tests indicated that there were the anticipated significant sex differences for lean body mass, body fat percentage, and handgrip strength [51] (see Table 5). Within this study, relationships between cognitive flexibility and the health status parameters were also assessed separately for the males and females. For the males, there was no significant (*p* > 0.05) relationship between cognitive flexibility (derived from the CNS VS) and body fat percentage (r = 0.00; *p* = 0.99), strength (r = −0.15; *p* = 0.60), or upslope (r = 0.02; *p* = 0.94). Similarly, for the females, there was no significant relationship between the objectively measured degree of cognitive flexibility and body fat percentage (r = −0.08; *p* = 0.77), strength (r = 0.47; *p* = 0.08), or upslope (r = −0.04; *p* = 0.08).

## 4. Discussion

This was the first study to examine both self-reported and objectively measured levels of cognitive flexibility to determine the proclivity of individuals to various exercise programs as well as their resulting health status. Specifically, our purpose was two-fold: (i) investigate the influence of self-reported cognitive flexibility on exercise perceptions and choice, and (ii) examine the relationship between objectively measured cognitive flexibility and CVD risk factors. Our hypotheses were that individuals’ perceptions of exercise are influenced by their level of cognitive flexibility, and a mismatch between one’s flexibility and the demands of an exercise program may lead to biased perceptions of the program. Further, we investigated if flexibility is related to, or perhaps predictive of, CVD risk. Our principal findings were: (a) individuals’ self-reported flexibility does influence perceptions of exercise efficacy and the effort required for an exercise program to be beneficial, as well as preference for certain exercise programs; however, (b) there was no measure of health status that was significantly related to objectively measured cognitive flexibility. Previous research has noted that self-report measures of executive cognitive abilities often have more variance accounted for by personality traits than objective cognitive performance, and this may be the case with the cognitive flexibility construct as well, as discussed below [52].

The results of Study 1 demonstrated that individuals intuitively seek out health-based programs that align with their self-reported level of cognitive flexibility. This finding suggests that, when given a choice, individuals should choose programs in which they have a higher likelihood of success. Our findings are consistent with existing research that suggests that, in decision-making contexts, one’s degree of flexibility can influence the evaluation of attributes and features of choices that facilitate choosing an option that aligns with their degree of cognitive flexibility [28,29]. Further, when assessing a commercial exercise program with a rigid structure, individuals who exhibit high flexibility perceive the program as requiring more effort to be successful. We reason that this perception may be due to a mismatch between one’s flexibility and the program demands. Relatedly, the perceived effort had a positive, significant relationship with the perceived effectiveness, which is consistent with a well-documented lay theory of more effort leading to more success [53].

As previously noted, self-control performance decreases in mismatching contexts; thus, individuals may perceive themselves as working harder but objectively finding less success. Navigating this landscape of exercise choice can be challenging, as individuals do not always have choices that are clear in being framed as “flexible” or “rigid”, as they are here; however, it seems that, if given the opportunity, individuals will seek out a program that matches their flexibility. If an individual finds alignment (i.e., a match), it is possible that they will experience greater enjoyment and perhaps, as a result, greater motivation. This would collectively lead to greater adherence or reduced ‘dropout’, [17]. Paradoxically, however, individuals may find these programs to require less effort, which challenges a deeply ingrained lay theory associating more effort with more effectiveness. Practitioners should be mindful of this paradox and seek to encourage individuals to find a program “match” and explain that engagement in such a program—while seemingly requiring less effort—may realistically result in greater adherence and effectiveness (vs. a program “mismatch”). Collectively, we interpret the findings from Study 1 to suggest a new paradigm of exercise *choice*, rather than fixed exercise *prescription*.

Through this investigation, we also aimed to better understand why individuals may not engage in adequate levels of physical activity and exercise or successfully adhere to programs designed to improve their health status. Through the identification of how flexibility influences perceptions of exercise programs, improvements in current behavioral-based exercise interventions can be made and, perhaps, even foster future adherence [15,17]. Many current exercise interventions are ineffective. For example, providing information concerning the consequences related to unhealthy behavior (e.g., a sedentary lifestyle can lead to obesity/heart disease) is insufficient to motivate lasting behavioral change (e.g., adherence to regular physical activity) [54]. Additionally, commercial weight loss and exercise products (e.g., WeightWatchers) vary in their efficacy (e.g., reduced CVD risk) and user adherence remains tenuous [16]. Previous investigations have confirmed that structured (favors rigidity) and unstructured (favors flexibility) exercise interventions are both capable of yielding meaningful health benefits [55,56]. Dunn et al. [55] reported that lifestyle (flexible) and structured (rigid) physical activity interventions improved CVD risk factors to a similar extent. Additional indirect evidence provided by Viken et al. [57] suggested that, in older adults randomized to a structured, rigid workout plan (supervised, scheduled, mandatory attendance) versus a more flexible workout (unsupervised, open-ended scheduling) regimen, flexibility was a significant predictor of attrition. Furthermore, 20.3% of participants in the supervised exercise dropped out, whereas only 9.4% quit participating in the unsupervised group. Although the authors [57] did not provide a measure of cognitive flexibility, it is reasonable to hypothesize that a match between the demands of an exercise prescription and an individual’s degree of cognitive flexibility should encourage behavior changes, such as increasing adherence [54]. Thus, it remains possible that a mismatch between individuals’ cognitive flexibility and the results of randomization (e.g., being assigned to a flexible vs. rigid group) can have a marked impact on adherence [57], highlighting a real need to continue investigating whether prescribing exercise that mismatches one’s cognitive flexibility can lead to faulty perceptions and expectations, thus affecting adherence.

To our surprise, in Study 2, there were no significant associations observed between health status and objectively measured cognitive flexibility. Due the documented link between flexibility and participating regularly in physical activity and exercise [5,33] and the association between obesity and lower degrees of cognitive function [21,22,39,40], it was our assumption that individuals who displayed superior health would also exhibit greater cognitive flexibility. The current results, however, do not support this notion, and flexibility (rigidity) should not be assumed to predict reduced (increased) CVD risk for otherwise healthy adults. Although this finding was contrary to the stated hypotheses, it may be encouraging speculation that healthy, cognitively rigid individuals are not predisposed to a higher risk of CVD. Additionally, it is possible that results derived from measured versus self-reported cognitive flexibility may promote the current discrepancies in the associated literature. Regardless, it may still be appropriate for researchers to extend our findings to the practical application of considering an individual’s degree of cognitive flexibility (or rigidity), rather than relying solely on the current health status of the patient/client, when attempting to mitigate attrition rates [5,33]. This approach may maximize enjoyment (e.g., by decreasing effort) and, therefore, the impact of the exercise prescription via suspected improvements in intervention adherence [15,17,58]. 

The current work is not without its limitations, and readers should consider the following aspects when evaluating the significance of our reported findings. First, as previously mentioned, cognitive flexibility is a complex cognitive function made up of several processes, and previous research has attempted to provide a unifying account that best explains the construct [25,33,59]. In line with this approach, we employed two measures aiming to operationalize cognitive flexibility, including one neuropsychological (objective measure; CNS Vital Signs) assessment and a self-report measure (Actively Open-Minded Thinking Scale). Although cognitive flexibility may be comprised of certain executive functions that are assessed by performance on the Stroop (i.e., inhibition) or SAT (i.e., set-shifting) tasks, there are other executive functions and mechanisms (e.g., updating) that may also appreciably relate to the observed differences in flexibility. That is, although the CNS Vital Signs measure indeed provides a reliable assessment of cognitive flexibility, the self-report measure employed here may have provided additional relevant and unique insights related to exercise perception and choice, including aspects of personalities [52]. Future research could consider other behavioral measures, such as a mental categorization task [60], other self-report measures [45,46,61], or even a measure of psychological flexibility [62] to extend the reach of this framework. Secondly, as stated above, we recruited participants from university student populations, which limits the generalizability to other individuals. Based on body composition, vascular responses, and handgrip values, these individuals were above average in terms of health status, which limits the scope of our reported relationships. It remains very likely that our lack of association between cognitive flexibility and health status was largely influenced by the adequate health of our college-aged sample. We acknowledge that using clinical or other particular populations (e.g., older adults) may have yielded different associations, but based on the lack of current related data, it was our intention to provide a foundation for this specific research agenda. Perhaps, individuals of lesser health (e.g., exhibiting signs and symptoms of CVD risk factors) would present relationships among vascular function, handgrip strength, body composition, and cognitive flexibility. Aligned with age-related decreases in health, it is widely accepted that cognitive flexibility decreases as well. Thus, future work should determine whether improved health (i.e., a reduction of CVD risk factors) leads to a slower decline in cognitive flexibility. Relatedly, while habitual exercise is clearly beneficial for cognition [3,9,13,18], more research is needed to determine the type and dosage of physical activity that is most effective when considering one’s degree of flexibility, especially within particular populations, including clinical and aging individuals [63]. These associated future studies will also be able to address whether there are differences in exercise perceptions (e.g., enjoyment) and CVD risk factors among individuals with varying degrees of cognitive flexibility. Overall, our currently reported data should provide rationale and direction for future high-impact investigations.

## 5. Conclusions

The current investigation highlighted how cognitive flexibility influences perceptions of exercise programs, but did not support the hypothesis that there are relationships among risk factors of CVD indicative of health status and cognitive flexibility for healthy college-aged adults. It is our current interpretation that these findings provide a new perspective on the role of flexibility in predicting an individual’s decision making and perceptions of performance in exercise contexts. Now, it is our working hypothesis that cognitive flexibility could be a valuable construct to predict individuals’ choice of, and subsequent adherence to, prescriptions targeted at improving overall health, especially in particular populations, and specifically with self-report approaches. In other words, we propose that exercise prescriptions targeted at reducing the risk of CVD should include a two-pronged approach to facilitate adherence: prescribing exercise regimens that are both actionable (based on health status) and desirable (based on degree of cognitive flexibility).

## Figures and Tables

**Table 1:** Correlations between sex and the means of cognitive flexibility measures in each study.

	Male	Female	Correlation Coefficient	*p*-Value
Study 1—AOT	4.9 ± 0.8	4.9 ± 0.6	*r* = −0.019	*p* > 0.70
Study 2—CNS Vital Signs	101.3 ± 17.5	98.9 ± 21.0	*r* = −0.064	*p* > 0.70

**Table 2:** The mean ± SD values corresponding to the variables used for correlational analyses in Study 2.

	Study 2		
	Mean	SD	n
CNS Vital Signs Cognitive Flexibility	100.1	19.0	30
Lean Body Mass (kg)	126.1	27.3	30
Fat Mass (kg)	39.8	5.2	30
Body Fat (%)	24.2	5.2	30
Handgrip (kg)	37.3	12.1	30
Upslope (%∙s^−1^)	2.1	0.7	30

Note: Health status parameter values were averaged across visits.

**Table 3:** Complete correlation matrix of specific markers of health status and cognitive flexibility in Study 2.

n = 30	Weight (kg)	Body Mass Index	Lean Body Mass (kg)	Fat Mass (kg)	Body Fat (%)	Strength (kg)	Cognitive Flexibility (CNS VS)	Upslope (%∙s^−1^)
**Weight (kg)**	**1.00**							
Body Mass Index	**0.882**	1.00						
Lean Body Mass (kg)	**0.796**	**0.642**	1.00					
Fat Mass (kg)	0.241	**0.435**	0.262	1.00				
Body Fat (%)	−0.358	−0.091	**−0.441**	**0.735**	1.00			
Strength (kg)	**0.754**	**0.610**	**0.907**	0.181	**−0.465**	1.00		
Cognitive Flexibility (CNS VS)	−0.018	−0.115	0.009	0.063	0.059	−0.036	1.00	
Upslope (%∙s^−1^)	0.167	0.054	0.087	−0.262	−0.352	0.113	0.020	1.00

Note: Bolded values indicate a significant (*p* < 0.05; two-tailed) Pearson correlation coefficient.

**Table 4:** Test–Retest Reliability and Consistency Results.

	Study 2: (2,1 Model; ICCs), Systematic Error (Repeated Measures ANOVA), Coefficient of Variation (CV; Normalized Absolute Reliability)					
	Visit 1	Visit 2		ICC	*p*-Value	CV
Handgrip Strength (kg)	37.0 ± 11.6	38.3 ± 12.3		0.95	0.06	6.73
Upslope (%∙s^−1^)	2.31 ± 0.9	2.06 ± 0.7		0.56	0.18	29.7
Lean Body Mass (kg)	59.2 ± 12.9	58.5 ± 12.1		0.98	0.19	2.87
Fat Mass (kg)	20.0 ± 10.3	20.6 ± 11.2		0.98	0.09	6.99

**Table 5:** Participant characteristics in Study 2.

n = 30	Male	Female	*p*-Value
Age (yr)	23 ± 1	22 ± 1	*p* > 0.05
Weight (kg)	85.0 ± 11.6	64.9 ± 9.9	***p* < 0.05**
Height (cm)	176.5 ± 6.3	166.9 ± 7.2	***p* < 0.05**
BMI	27.3 ± 3.6	23.2 ± 2.6	***p* < 0.05**
MAP (mmHg)	89.5 ± 13.2	85.2 ± 14.1	*p* > 0.05
Lean Body Mass (kg)	66.9 ± 9.5	47.5 ± 5.2	***p* < 0.05**
Fat Mass (kg)	18.4 ± 5.3	17.8 ± 4.5	*p* > 0.05
Body Fat (%)	21.4 ± 4.8	27.0 ± 3.9	***p* < 0.05**
Grip Strength (kg)	46.6 ± 9.3	28.2 ± 5.8	***p* < 0.05**
Upslope (%∙s^−1^)	2.20 ± 0.7	2.01 ± 0.8	*p* > 0.05
Systolic Blood Pressure	127.6 ± 72.7	120.9 ± 11.2	*p* < 0.05
Diastolic Blood Pressure	72.7 ± 8.4	72.0 ± 9.8	*p* > 0.05

Note: Bolded values denote significance.

## Data Availability

The authors did not preregister their research with an institutional registry.
